# Mechanisms of ferroptosis and targeted therapeutic approaches in lymphoma

**DOI:** 10.1038/s41419-023-06295-w

**Published:** 2023-11-25

**Authors:** Tiantian Yu, Zijun Y. Xu-Monette, Li Yu, Yong Li, Ken H. Young

**Affiliations:** 1 Hematopathology Division and Department of Pathology, Duke University Medical Center, Durham, NC USA; 2 Department of Hematology and Oncology, The Second Affiliated Hospital of NanChang University, Nanchang, China; 3 Department of Medicine, Baylor College of Medicine, Houston, TX USA; 4 Duke Cancer Institute, Durham, NC USA

**Keywords:** B-cell lymphoma, Cell death

## Abstract

Lymphoma is the sixth most common type of cancer worldwide. Under the current treatment standards, patients with lymphoma often fail to respond to treatment or relapse early and require further therapy. Hence, novel therapeutic strategies need to be explored and our understanding of the molecular underpinnings of lymphomas should be expanded. Ferroptosis, a non-apoptotic regulated cell death, is characterized by increased reactive oxygen species and lipid peroxidation due to metabolic dysfunction. Excessive or lack of ferroptosis has been implicated in tumor development. Current preclinical evidences suggest that ferroptosis participates in tumorigenesis, progression, and drug resistance of lymphoma, identifying a potential biomarker and an attractive molecular target. Our review summarizes the core mechanisms and regulatory networks of ferroptosis and discusses existing evidences of ferroptosis induction for the treatment of lymphoma, with intent to provide a framework for understanding the role of ferroptosis in lymphomagenesis and a new perspective of lymphoma treatment.

## Facts

- Ferroptosis is an iron-dependent type of regulated cell death characterized by increased reactive oxygen species and excessive lipid peroxidation.
- The p53, MYC, and PI3K pathways that are often dysregulated in lymphoma also regulate ferroptosis.
- Crosstalk between ferroptosis and other regulated cell death pathways can compound the role of ferroptosis in lymphoma.
- Ferroptosis dysregulation is involved in lymphomagenesis of some lymphoma subtypes.
- Promising preclinical studies show that lymphoma cells are susceptible to ferroptosis induction, yet further investigation is warranted.

## Introduction

**Fig. 1:**
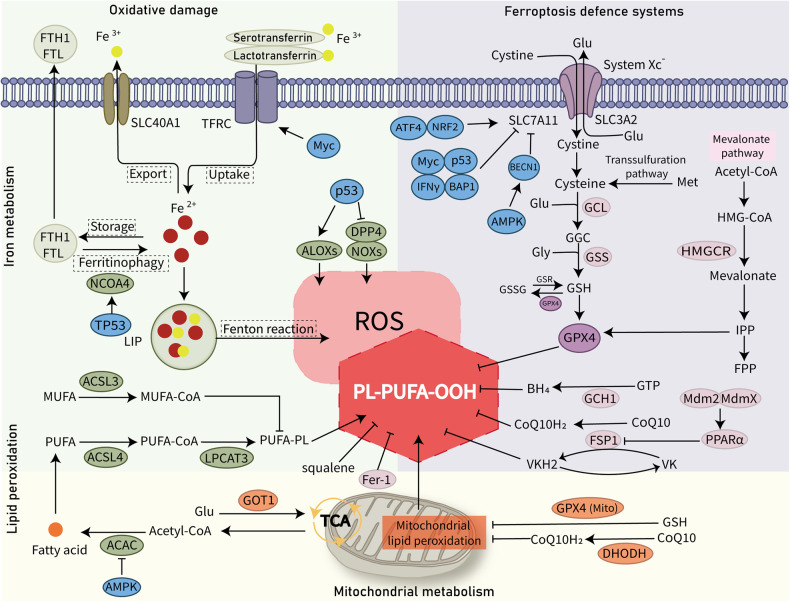
Molecular mechanisms of ferroptosis. The core mechanism underlying the induction of ferroptosis is iron-dependent lipid peroxidation, which leads to the accumulation of reactive oxygen species (ROS). The iron metabolism involved in ferroptosis induction is regulated by several proteins, including ferritin components (FTH1, FTL), solute carrier family 40 member 1 (SLC40A1), serotransferrin, lactotransferrin, and transferrin receptor (TFRC). Acetyl-CoA carboxylase (ACAC)-mediated fatty acid synthesis increases accumulation of polyunsaturated fatty acids (PUFA) and promotes the formation of polyunsaturated fatty acid-containing phospholipids (PUFA–PL) via fatty acid–CoA ligase 4 (ACSL4) and lysophosphatidylcholine acyltransferase 3 (LPCAT3). Monounsaturated fatty acids (MUFA) are resistant to oxidation and inhibit PUFA-PL peroxidation. The glutathione (GSH)/glutathione peroxidase 4 (GPX4) antioxidant system initiated by system Xc^−^ is the main cellular defense against ferroptosis. Other independent defense mechanisms include the CoQH2 system and other antioxidants such as tetrahydrobiopterin/dihydrobiopterin and squalene. FSP1 and mitochondrial DHODH reduce CoQ to CoQH_2_ and suppress ferroptosis. ACSL3 fatty acid–CoA ligase 3, ALOXs arachidonate lipoxygenases, BECN1 beclin 1, BH4 tetrahydrobiopterin, CoQ10 coenzyme Q10, DDP4 dipeptidyl peptidase-4, DHODH dihydroorotate dehydrogenase, Fer-1 Ferrostatin 1, FSP1 ferroptosis suppressor protein 1, GCH1 GTP cyclohydrolase-1, GCL glutamate-cysteine ligase, Glu glutamic acid, GSR glutathione-disulfide reductase, GSS glutathione synthetase, GSSG glutathione disulfide, oxidized glutathione, GTP guanosine triphosphate, IFN-γ interferon-γ, LIP labile iron pool, NCOA4 nuclear receptor co-activator 4, NOXs NADPH oxidases, PL-PUFA-OOH polyunsaturated fatty acid-containing phospholipids hydroperoxide, TCA tricarboxylic acid cycle, VK vitamin K, VKH2 vitamin K hydroquinone.

Ferroptosis is an iron-dependent form of regulated cell death (RCD) first defined in 2012 [1], and differs in morphology, biochemistry, and immune features from other forms of RCD, such as apoptosis, necroptosis, and pyroptosis [2]. The biochemical mechanisms underlying ferroptosis involve lipid peroxidation and intracellular iron accumulation, which in turn result in the production of lethal reactive oxygen species (ROS) and alkyl oxygen radicals, inducing membrane damage and disorganization [3]. Broadly, ferroptosis can be triggered through two pathways [4]: the extrinsic and intrinsic pathways. The extrinsic pathway is a receptor-dependent pathway primarily triggered by the activation of iron transporters or inhibition of cell membrane transporters in the cystine/glutamate (Glu) antiporter system Xc^−^. The intrinsic pathway is mediated by regulation of intracellular antioxidant enzymes, such as glutathione peroxidase 4 (GPX4) [5] and those in metabolism pathways of amino acids, iron, and polyunsaturated fatty acids (PUFA). Of note, the PUFA pathway is perhaps the most important metabolic pathway in ferroptosis (Fig. 1), as PUFA can interact with peroxides to produce toxic lipid peroxides (PL-PUFA-OOH) under quiescent conditions [6]. Conversely, toxic PL-PUFA-OOH can be reduced by GPX4 into non-toxic products [7].

**Fig. 2:**
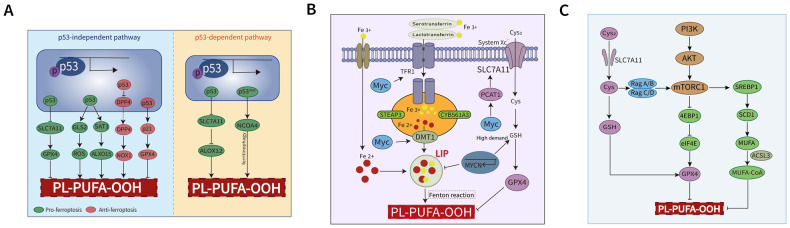
Lymphoma-related pathways are involved in ferroptosis regulation. **A** The role of p53 in ferroptosis. p53 promotes ferroptosis by regulating solute carrier family 7 member 11 (SLC7A11), glutamine synthase 2 (GLS2), and spermidine/spermine N1-acetyltransferase 1 (SAT1)/arachidonate lipoxygenase 15 (ALOX15), and inhibits ferroptosis by targeting p21 and blocking DPP4 activity in a p53-independent and transcription-independent manner. Meanwhile, the p53 pro-ferroptosis role can be mediated by arachidonate lipoxygenase 12 (ALOX12) and NCOA4-mediated ferritinophagy via a p53-dependent pathway. **B** The regulatory role of Myc in ferroptosis. Myc regulates ferroptosis via TFR1, DMTQ, and PCAT1. Oncogenic MYCN activity sensitizes cells to ferroptosis upon cyst(e)ine depletion, caused by decreased GSH and consequent massive lipid peroxidation. **C** The PI3K–AKT–mTOR signaling pathway suppresses ferroptosis. PI3K–AKT–mTOR signaling synergizes with the SLC7A11–GSH–GPX4 pathway by upregulating SREBP1 and SCD1 promoting MUFA synthesis, and also augments GPX4 protein synthesis by inhibiting eukaryotic translation initiation factor 4E (eIF4E)-binding protein 1 (4EBP1). Cys cysteine, Cys2 cystine, DMT1 divalent metal transporter 1, PCAT1 peptidase-containing ABC transporter 1, SREBP1 sterol regulatory element-binding protein 1, STEAP3 six-transmembrane epithelial antigen of the prostate 3, SCD1 stearoyl-CoA desaturase-1, TFR1 transferrin receptor 1.

Noteworthy progress has been made in our understanding of the role of ferroptosis in the molecular pathogenesis of many cancers. There is crosstalk between ferroptosis and multiple cancer-associated signaling pathways in cancer cells. For example, the tumor suppressor p53, mutated in over 50% of tumors, may promote or suppress ferroptosis in oxidative/metabolic stress conditions (Fig. 2A) [8]. By contrast, the PI3K–AKT–mTOR pathway inhibits ferroptosis via promoting GPX4 protein synthesis [9] or by regulating lipid metabolism (Fig. 2B). On one side, oncogenic signaling-mediated activation of ferroptosis has been implicated in promoting tumor growth, immune evasion, tumor progression, and therapeutic resistance [10]. On the other side, high burden of ROS, metabolic alterations, and specific mutations render cancer cells susceptible to ferroptotic cell death [11, 12]. These findings suggest that ferroptosis plays diverse roles in cancer development and therapeutic responses of tumors [13, 14]. Thus, modulating ferroptosis and targeting cancer vulnerabilities could have great therapeutic efficacy, especially when combined with conventional treatments [15–17].

Recent studies have demonstrated that lipids are associated with cancer development and are elevated in lymphoma. Choline metabolism, which is a crucial component of lipid synthesis, is dysregulated in lymphoma [18]. Besides, there is a hypothesis that lymphoma dependent on lipoprotein-mediated cholesterol uptake is susceptible to ferroptotic cell death [19]. However, to date, few studies have examined ferroptosis in lymphoma. As research on ferroptosis increases, it is important to improve our understanding of ferroptosis in lymphoma, especially in the subsets with clinical needs. This review aimed to provide a comprehensive framework for the regulatory networks involved in ferroptosis in lymphoma and targeting ferroptosis as a therapeutic strategy for lymphoma based on the findings of the aforementioned studies.

## Oxidative damage inducing ferroptosis is implicated in lymphoma suppression

### lipid peroxidation control and ferroptosis regulation

Lipid peroxidation is the primary feature of ferroptosis, and the main mechanism is the peroxidation of PUFA and the fatal accumulation of lipid peroxides (Fig. 1). A PUFA-rich diet has been reported to suppress tumor growth and prolong survival in a mouse tumor model of colorectal carcinoma [20]. Lipid peroxidation has been implicated in cancer progression and is the driving force of ferroptotic cell death in germinal center B cell-like (GCB) diffuse large B-cell lymphoma (DLBCL) [21]. Acyl-CoA synthetase long chain family member 4 (ACSL4) and lysophospholipid acyltransferase 5 encoded by the lysophosphatidylcholine acyltransferase 3 (*LPCAT3*) gene are essential enzymes that regulate the biosynthesis and remodeling of PUFAs [22, 23]. ACSL4 catalyzes the conversion of free PUFAs to activated coenzyme-A (CoA)-PUFAs [22], and LPCAT3 catalyzes their esterification to form PUFA-PLs [24]. PUFA-PLs are susceptible to oxidation by PL-PUFA-OOH, which induces ferroptosis [25, 26]. Accordingly, knockdown of *ACSL4* or *LPCAT3* in vitro blocks the peroxidation of PUFA-PLs and prevents or attenuates ferroptosis [22, 27]. In addition, some membrane proteins such as cytochrome P450 oxidoreductase [28], NADPH oxidase [29], and arachidonate lipoxygenase (ALOX) [30] increase lipid peroxidation, which induces production of ROS. In contrast, monounsaturated fatty acids (MUFA) are converted to MUFA-CoA by fatty acid–CoA ligase 3 (ACSL3), which inhibits the peroxidation of PUFA-PLs and in turn inhibits ferroptosis [31]. Ferrostatin-1 exerts an anti-ferroptotic role by inhibiting the activity of lipid hydroperoxides [32]. Moreover, AMPK interferes with the phosphorylation of acetyl-CoA carboxylase (ACAC), which in turn inhibits PUFA production and consequently ferroptosis [33]. These data imply that lipid peroxidation is an essential factor that regulates the development and progression of cancers including lymphoma and maybe a potential new target.

### Regulation of iron metabolism in lymphoma

**Fig. 3:**
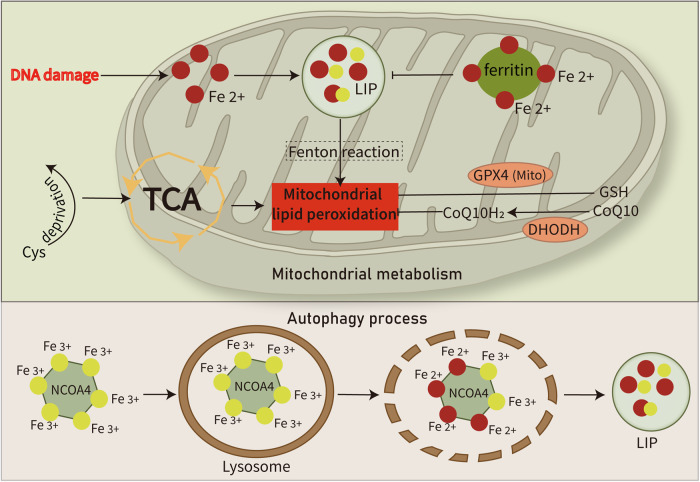
Mitochondrial metabolism and autophagy regulate ferroptosis. (Top) Mitochondrial metabolism plays an indispensable role in ferroptosis regulation by mitochondrial ferritin-mediated iron chelation, cysteine (Cys) deprivation-induced TCA cycle stimulation, and DHODH-mediated ferroptosis defense. (Bottom) Autophagy, a cell survival mechanism supporting cancer cell metabolism and survival under stress, induces autophagy-dependent ferroptosis in cancer cells. This phenomenon is marked by increased intracellular iron concentration and lipid peroxidation driven by hyperactivated NCOA4-mediated autophagy. CoQ10 coenzyme Q10, DHODH dihydroorotate dehydrogenase, GSH glutathione, LIP labile iron pool, NCOA4 nuclear receptor co-activator 4, TCA tricarboxylic acid cycle.

Typically, normal cells regulate iron uptake, utilization, storage, and export to maintain the iron pool balance, whereas malignant cells can be observed as iron unbalanced. Iron, a trace element, is required for the growth of malignant tumors, such as Burkitt lymphoma (BL) [34] and T-cell lymphoma [35]. Consistently, a case report described a patient with follicular lymphoma (FL) characterized by hyperferritinemia and widespread iron deposition [36]. Abnormal iron homeostasis has been implicated in the biogenesis and development of lymphoma, leading to an imbalance in the intracellular labile iron pool (LIP) level (Fig. 1). High LIP levels result in increased peroxidation in lymphoma cells [37], which promotes ferroptosis. Most intracellular iron exists in a free state as Fe^2+^. Fe^2+^ reacts with hydrogen peroxide via the Fenton reaction to generate ROS. Ferritin, an iron storage protein, stores iron in the form of Fe^3+^, thereby inhibits the Fenton reaction. Iron uptake mediated by transferrin receptor (TFRC/TFR) [38], lactotransferrin (LTF) [39] and serotransferrin (STF) [40] enhances ferroptosis, whereas solute carrier family 40 member 1 (SLC40A1) inhibits ferroptotic cell death by facilitating iron export [41]. Furthermore, the interaction of nuclear receptor co-activator 4 (NCOA4) and ferritin heavy chain 1 (FTH1) delivers iron-bound ferritin to autophagosomes for lysosomal degradation, which is termed as ferritinophagy [42]. This process releases iron from ferritin to the LIP [43, 44]. Interestingly, the lymphoma-associated *MYC* oncogene is involved in iron metabolism and can increase intracellular LIP by activating transferrin receptor 1 (TFR1) (Fig. 3). The modification of iron metabolism by c-Myc can disrupt iron homeostasis and affect lymphoma growth [45]. Consequently, targeting iron homeostasis represents a new therapeutic strategy for patients with lymphoma, especially Myc-driven lymphoma.

## Antioxidant defense systems protect lymphoma cells from ferroptosis

### GPX4-dependent pathways

GPX4, an antioxidant enzyme, mitigates lipid peroxidation in lymphoma and hence is a central repressor of ferroptosis [46]. GPX4 plays an essential role in the growth and development of innate-like B cells (such as B1 and marginal zone B cells) and antibody responses of follicular B2 cells [47]. Vitamin E cooperates with GPX4 to maintain the lipid redox balance and prevent ferroptosis in hematopoietic stem and progenitor cells [48]. High GPX4 expression is negatively correlated with clinical outcomes in a study in DLBCL [49]. The activity of GPX4 is regulated by the level of glutathione (GSH) synthesized from cysteine, glycine, and Glu, which alleviates oxidative stress by eliminating ROS [46]. GSH can be transformed into oxidized glutathione (GSSG), which is then reduced to GSH to maintain a steady state. Notably, the system Xc^−^, which is comprised of solute carrier family 3 member 2 (SLC3A2) and solute carrier family 7 member 11 (SLC7A11), is integral to GSH production. The system Xc^−^, an antiporter on the cell membrane, transports one cystine (oxidized derivative of cysteine) to the intracellular space for subsequent GSH production and releases one Glu into the extracellular space [50]. The SLC7A11–GSH–GPX4 axis is the primary cellular defense mechanism against ferroptosis (Fig. 1) which is positively regulated by NRF2 [51] and ATF4 [52] and negatively regulated by the suppressor genes p53 [53] and BAP1 [54]. Moreover, the phosphorylation of beclin 1 (BECN1) can suppress the activity of SCL7A11, leading to ferroptotic cell death in an AMPK-mediated manner [55].

Pharmacological inhibitors of system Xc^−^ (erastin, imidazole ketone erastin [IKE], and sulfasalazine) or GPX4 (RSL3 or ML-210) induce ferroptotic cell death, and present attractive therapeutic strategies for clinical application [56]. IKE has been identified as an effective inhibitor of system Xc^−^ in mouse models of lymphoma [57]. On the other hand, several cell lines with functionally inactive GPX4 are resistant to ferroptosis inhibitors, suggesting that the involvement of mechanisms other than the SLC7A11-GSH-GPX4 dependent pathway. Although these inhibitors show high responsiveness, they are not suitable for clinical use due to the toxicity. Gpx4 knockout triggers acute renal failure in mice [58] which can be rescued by ACSL4 inhibitors [22], whereas Lpcat3 knockout is also lethal in vivo, likely because LPCAT3 is essential for membrane phospholipid composition, triglyceride transport, and lipoprotein production [59–61].

### GPX4-independent pathways

Recent studies have identified that the ubiquinol (CoQH_2_) system is a GPX4-independent defense mechanism against ferroptosis [62, 63]. The GPX4 and CoQH_2_ systems are further organized into constituents located in the non-mitochondrial (cytosolic GPX4 in the GPX4 system and ferroptosis suppressor protein 1 [FSP1] in the CoQH_2_ system [62, 64]) and mitochondrial compartments (mitochondrial GPX4 in the GPX4 system and mitochondrial dihydroorotate dehydrogenase [DHODH] in the CoQH_2_ system) (Fig. 1). FSP1, previously known as AIFM2, is an oxidoreductase that reduces coenzyme Q10 (CoQ10, also known as ubiquinone-10) to CoQ10H_2_ using NAD(P)H [62]. The FSP1–CoQ10–NAD(P)H pathway is a self-contained system parallel to and in concert with the SLC7A11–GSH–GPX4 axis to suppress ferroptosis [63]. Vitamin K (more specifically, the reduced form hydroquinone) can protect against detrimental lipid peroxidation via FSP1-mediated reduction [65, 66]. In addition, MDM2 and MDMX can decrease the protein levels of FSP1 and promote ferroptosis by activating PPARα in a p53-independent manner [67]. The DHODH-CoQH_2_ pathway is a mitochondrial CoQH2 system that acts in parallel to the mitochondrial GPX4 pathway against ferroptosis. When mitochondrial GPX4 is knocked down, DHODH inhibits lipid peroxidation in the mitochondria [68]. Consequently, inactivation of mitochondrial GPX4 and DHODH increases mitochondrial lipid peroxidation and stimulates ferroptosis.

Other GPX4-independent ferroptosis-defense mechanisms include the GTP cyclohydrolase-1–tetrahydrobiopterin-phospholipid pathway [69] and squalene (Fig. 1). Squalene is an antioxidant against lipid peroxidation and ferroptosis, and was identified by Garcia-Bermudez et al. as a metabolite in the cholesterol synthesis pathway abnormally accumulated in cholesterol auxotrophic ALK+ anaplastic large cell lymphoma (ALCL) cells conferring a growth advantage under oxidative stress [27]. As cholesterol is metabolically required by human cells, growth of ALK + ALCL cells is sensitive to cholesterol-depleting therapy or targeting the low-density lipoprotein receptor (LDLR) [27]. Squalene accumulated in the upstream cholesterol synthesis could be a pathogenic mechanism of ALK + ALCL and cause resistance to GPX4-inhibition treatment; inhibition of squalene sensitizes ALK + ALCL cells to GPX4 inhibitors ML162 and RSL3 [27]. Studies on the mechanisms underlying the GPX4-independent pathways will inspire ideas for treating patients who may not respond to GPX4 inhibition-based approaches.

## Lymphoma-related pathways are involved in ferroptosis regulation

### The p53 signaling pathway

Mutations in the *TP53* gene and consequently in p53 proteins result in inactivation of the p53 tumor suppressor pathway in cancers [70, 71]. *TP53* encodes p53, a tumor suppressor protein, which plays an important role in lymphoma tumorigenesis [72, 73]. *TP53* is commonly mutated in small lymphocytic lymphoma/chronic lymphocytic leukemia (SLL/CLL) [74, 75] and non-Hodgkin lymphoma (NHL) [72] including DLBCL [76], FL [77], mantle cell lymphoma (MCL) [78], and BL [79], and *TP53* mutations are associated with poor overall survival (OS) and progression-free survival (PFS). Recent studies have reported that *TP53* mutations can modulate the function of p53 in the context of various cellular process, such as antioxidant defense and ferroptosis [8]. For example, the P47S variant of p53 is associated with ferroptosis phenotypes and increased resistance to glutamate-mediated cytotoxicity [80], whereas the 3KR acetylation-defective variant of p53 is associated with defective p53-dependent induction of apoptosis and sensitivity to oxidative stress-dependent ferroptosis [53]. These reports suggest that the p53 pathway contributes to the regulation of ferroptosis.

p53 was first reported to mediate ferroptosis in a reduction/oxidation-dependent manner in 2015 [53]. p53 can efficiently repress the expression of SLC7A11, thereby enhancing the sensitivity of tumor cells to ferroptosis inducers, such as erastin. p53 also promotes ferroptosis by regulating metabolism-related genes, such as GLS2 [81], SAT1/ALOX15 [82], and FDXR [83], and non-coding RNAs, such as LINC00336/miR-6852 [84] and PVT1/miR-214 [85]. Different from the induction of ferroptosis by erastin or GPX4 inhibitors requiring ACSL4, p53-mediated ferroptosis under ROS stress requires ALOX12 and p53 activation [86]. In contrast, p53 inhibits ferroptosis through the target gene p21 [87] and blocking dipeptidyl-peptidase-4 (DPP4) activity [88]. DPP4 binds to the NADPH oxidase 1 (NOX1), and the DPP4-NOX1 complex promotes lipid peroxidation and ferroptosis [88]. Thus, DPP4 inhibitors limit the anti-cancer activity of ferroptosis inducers (such as erastin). Notably, the regulation of ferroptosis by p53 is likely to be dependent on intracellular ROS levels: p53 plays a protective role against ferroptosis when intracellular ROS levels are low, whereas p53 promotes ferroptosis when intracellular ROS levels are abnormally high [89]. Collectively, these reports highlight the dual context-dependent pro- and anti-ferroptosis roles of p53 (Fig. 2A).

In preclinical studies, the natural product kayadiol exhibited a significant anti-tumor effect via induction of the p53-dependent ferroptosis pathway in natural killer/T-cell lymphoma (NKTCL) [90]. Additionally, APR-246, a small molecule that reactivates the activity of p53 mutants, induces DLBCL cell death via ferroptosis, and triggers p53-dependent ferritinophagy in DLBCL cells with TP53 missense mutation on exon 7 [91]. These findings are crucial for understanding the mechanism underlying p53-mediated ferroptosis in lymphoma and provide a new direction for the treatment of p53-dependent lymphoma (Table 1).

**Table 1:** Summary of studies targeting the ferroptosis pathway in lymphoma.

Category	Agent	Target	Model	Mechanism	Ref
Ferroptosis inducer	DMF	GSH/GPX4	DLBCL cell lines	Increasing lipoxygenase expression and decreasing GSH and GPX4 expression	[21]
	Artesunate	GSH/GPX4	DLBCL cell lines; BL cell lines and xenograft model	Downregulating GSH and GPX4 expression and insensitive to ROS and lipid peroxidation	[145]
	ML-210	GPX4	Primary B Cell infected with B95.8 EBV	Inactivating GPX4 and inducing lipid ROS and cell death	[152]
	HDL NPs	SCARB1/GPX4	BL, DLBCL cell lines; DLBCL xenograft model	Binding SCARB1 to abolish GPX4	[19]
	Sulfasalazine	system Xc^−^	DLBCL cell lines and xenograft model	Reducing cystine uptake and cysteine supply	[143]
	IKE	system Xc^−^	DLBCL xenograft model	Inhibiting system Xc^−^ and leading to lipid peroxidation	[57]
	Erastin	SLC7A11	Primary B Cell infected with B95.8 EBV	Inhibiting SLC7A11 expression and restricting the supply of cysteine induces lipid ROS	[152]
	APR-246	TP53/NCOA4	TP53-mutated DLBCL cells and xenograft models	Reactivating the transcriptional activity of p53 mutants and enhance NCOA4	[91]
	Bortezomib	Noxa	MCL cell lines and primary tumor cells	Upregulating Noxa inducing mitochondrial depolarization and ROS generation	[149]
Ferroptosis inhibitor	Kayadiol	SLC7A11/GPX4	NKTCL cell lines	Targeting the SLC7A11/GPX4 axis reduces ROS accumulation	[90]
	Ironomycin	iron	DLBCL cell lines	Decreasing the intracellular labile iron pool	[96]

*DMF* dimethyl fumarate, *GSH* glutathione, *GPX4* glutathione peroxidase 4, *ROS* reactive oxygen species, *HDL-NPs* high–density lipoprotein-like nanoparticles, *SCARB1* scavenger receptor type B1, *IKE* imidazole ketone erastin.

### *MYC* signaling pathway

The oncogenic transcription factor *MYC* plays extensive roles in cancers including lymphoma, and its deregulation is associated with lymphoma progression and poor prognosis [92]. *MYC-IGH* and *MYC-IGL* translocations occur in 80% and 10% of BL cases, respectively [93]. Wang et al. speculated that Epstein-Barr virus (EBV)-infected BL is associated with iron deficiency because of its unique dependence on the ferrireductase CYB561A3 pathway [34, 94], suggesting a strong link between tumorigenesis and iron metabolism. The MYC family member MYCN was reported to mediate cysteine addiction, and make the fast proliferating cells vulnerable to cyst(e)ine depletion, inducing MYCN-dependent ferroptosis [95]. Although *MYC* translocation is uncommon in DLBCL, overexpression of Myc is observed in 30% of DLBCL cases. Recently studies found that *MYC* regulates downstream target genes involved in iron metabolism and the oxidative stress response (Fig. 2B). MYC can regulate several genes involved in iron metabolism, such as *TFRC* and the ferrous iron transporter *DMT1*, which promote ferroptotic cell death by increasing intracellular LIP [34, 45]. Thus, iron chelators or ironomycin treatment can effectively target Myc and TFR1, demonstrating a potential therapeutic strategy for lymphoma patients with Myc overexpression [96]. On the contrary, there are several reports that suggest that Myc can inhibit ferroptosis by interacting with PCAT1 and promoting SLC7A11 expression [97] or by downregulating KMD1A, thereby increasing malondialdehyde and iron levels [98]. Further understanding of the interplay between MYC and ferroptosis may improve the treatment of *MYC*-driven lymphoproliferative disorders.

### PI3K–AKT–mTORC1 signaling pathway

The PI3K–AKT–mTOR pathway is one of the most aberrantly active signaling pathways in various cancers [99]. Fatty acid synthase activity is positively correlated with the PI3K–AKT–mTOR pathway which promotes the oncogenic translation of DLBCL [100, 101]. mTOR complex 1 (mTORC1) maintains protein synthesis and cellular homeostasis [102] and also regulates ferroptosis [103]. Recent studies have elucidated two mechanisms by which mTORC1 suppress ferroptosis (Fig. 2C). First, mTORC1 plays a synergistic role with GPX4 to inhibit ferroptosis. Specifically, mTORC1 augments GPX4 synthesis by inhibiting eukaryotic translation initiation factor 4E-binding protienn1 (4EBP1) phosphorylation [9, 104]. Consistently, Torin1, an ATP-competitive mTOR inhibitor [105], promotes ferroptotic cell death by decreasing GPX4 protein levels [9]. Second, mTORC1 induces cancer cell resistance to ferroptosis by activating sterol regulatory element-binding protein 1 (SREBP1), which regulates the expression of stearoyl-CoA desaturase-1 (SCD1), an enzyme responsible for MUFA synthesis [106]. Accordingly, abnormal PI3K–AKT–mTORC1 signaling allows lymphoma cells to escape ferroptosis, which promotes tumor initiation and progression. Further studies should focus on simultaneously targeting both the mTOR pathway and ferroptosis. Treatment with mTOR inhibitors in combination with ferroptosis inducers could be a promising strategy for patients with lymphoma with intrinsic or acquired resistance.

## Mitochondrial metabolism regulating ferroptosis is high in lymphoma subset

Tumors rewire their metabolisms to ensure sufficient nutrients to support their growth. In this regard, cancer cell metabolism is tightly linked with cell death pathways through regulation of mitochondrial functions. OxPhos-DLBCL is a DLBCL subset identified by a gene signature involved in oxidative phosphorylation, mitochondrial metabolism, and the electron transport chain [107]. OxPhos-DLBCLs display enhanced mitochondrial energy transduction but are insensitive to inhibition of the B-cell receptor (BCR) signaling pathway [108]. Recently, mitochondria have also been reported to be involved in ferroptosis, apoptosis, and mitophagy [109, 110]. Mitochondrial metabolism regulates ROS generation and ferroptosis via three pathways (Fig. 3). First, mitochondrial ferritin chelates iron, inhibiting free iron accumulation, thereby blocking lipid peroxidation via the Fenton reaction [111]. DNA damage results in excessive free iron accumulation in the mitochondria, and increased lipid peroxidation of cell membranes, which ultimately induces ferroptosis [112]. Second, cystein deprivation stimulates the tricarboxylic acid cycle, which enhances mitochondrial respiration, increases lipid peroxidation and ROS production in the mitochondria and therefore promotes ferroptosis [109, 113]. Third, mitochondria possess a DHODH-mediated ferroptosis defense mechanism [68]. In the mitochondria, DHODH reduces CoQ10 to CoQ10H_2_, and this mechanism runs in parallel with mitochondrial GPX4 to inhibit ferroptosis. These findings suggest that the mitochondria play an essential role in ferroptosis regulation and these pathways are potential therapeutic targets for ferroptosis induction in lymphoma cells.

Mitophagy is a mitochondria targeted autophagy with controversial role in ferroptosis [109, 114]. Autophagy, a cellular mechanism that supports cancer cell metabolism and survival under stress [115], is generally thought to induce ferroptosis. Autophagy-dependent ferroptosis has been observed in cancer cells, and is characterized by intracellular iron concentration and lipid peroxidation caused by the hyperactivation of autophagy [116]. In addition, knockdown of autophagy-related genes can deplete intracellular iron and reduce lipid peroxidation, protecting cells from ferroptotic cell death [116]. NCOA4 is a selective cargo receptor for the lysosomal autophagic turnover of ferritin. NCOA4-mediated ferritinophagy promotes ferroptosis by increasing intracellular Fe^2+^ levels (Fig. 3) [44, 117]. Consistently, inhibition of NCOA4 [44] or proteasomal degradation targeted by HERC2 [42] can decrease ferritin degradation and suppress ferroptotic cell death. In turn, ROS can activate autophagy, which plays a significant role in ferritin degradation during ferroptosis [118]. These data contribute to a more profound understanding of the connection between ferroptosis and autophagy.

## Tumor microenvironment may interact with ferroptotic lymphoma cells

The tumor microenvironment (TME) plays complex roles in diverse processes of lymphoma, including pathogenesis, progression, drug resistance, and prognosis, by facilitating sustained tumor proliferation growth and sensitize lymphoma cells to immunotherapy [119, 120]. Cancer immunotherapy can restore and enhance the effector function of cytotoxic CD8^+^ T cells, thereby improving immune responses to tumor cells [120]. CD8^+^ T cell mediated-cytotoxicity is primarily mediated by perforin/granzyme- or Fas/Fas ligand signaling-induced apoptosis. Additionally, immunotherapy-activated CD8^+^ T cells secrete interferon-γ (IFN-γ), which downregulates the expression of SLC7A11 and SLC3A2 by targeting the JAK–STAT pathway. This in turn enhances lipid peroxidation and consequently induces ferroptosis in cancer cells [15]. Consistently, the anti-PD-L1 nivolumab can downregulate the expression of SLC3A2 and upregulate the expression of IFN-γ and CD8 as elucidated by transcriptome analysis. Blocking SLC7A11-mediated cystine uptake in combination with immunotherapy synergistically enhances CD8^+^ T cell-mediated anti-tumor activity and promotes ferroptotic cell death. Moreover, IFN-γ in combination with PUFA triggers ferroptosis and boosts anti-tumor immune responses dependent of ACSL4. Targeting the ACSL4 pathway may be more effective in increasing the efficacy of immunotherapy. Besides, ferroptotic cancer cells also release HMGB1 in an autophagy-dependent manner, which binds to AGER and promotes inflammatory responses in macrophages (however, the increased inflammatory TNF cytokine production was only confirmed at the transcription level) [121].

**Fig. 4:**
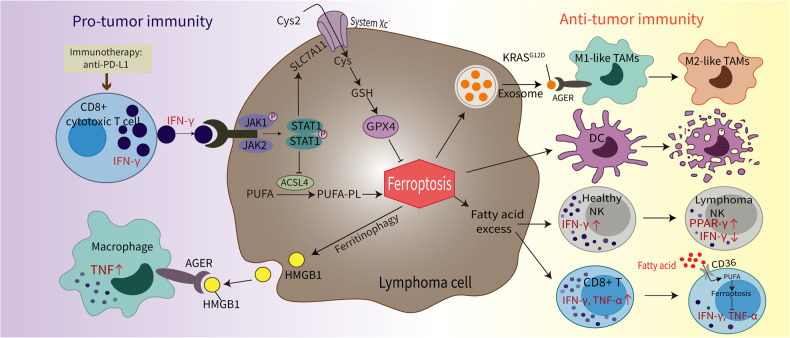
Relationship between ferroptosis and tumor immunity. CD8^+^ T cells induce ferroptotic cancer cell death by secreting interferon γ, which activates the JAK1–STAT1 signaling pathway, thereby regulating the SLC7A11–GSH–GPX4 axis. Ferroptotic cancer cells can release damage-associated molecular patterns (such as HMGB1) in a ferritinophagy-dependent manner, which binds to AGER to promote inflammatory response of macrophages. Autophagy-dependent ferroptotic cancer death also can release oncogenic KRAS^G12D^ protein, which triggers M2 macrophage polarization and hampers dendritic cell-mediated anti-tumor activities. Ferroptotic cancer cells also release fatty acids, which inhibit natural killer (NK) cell cytotoxicity and induce ferroptosis in CD8^+^ T cells by binding to CD36. Cys cysteine, Cys2 cystine, IFN-γ interferon-γ, GSH glutathione, GPX4 glutathione peroxidase 4, PPAR- γ peroxisome activator receptor γ, PUFA polyunsaturated fatty acids, PUFA–PL polyunsaturated fatty acid-containing phospholipids, TAM tumor associated macrophage, TNF-α tumor necrosis factor-alpha.

However, emerging evidence has also shown that ferroptotic cancer cells negatively affect immune responses. Ferroptotic cancer cells inhibit the maturation of dendritic cells (DCs) and disrupt antigen cross-presentation, thereby hampering DC-mediated anti-tumor activity [122]. In addition, autophagy-dependent ferroptosis of cancer cells release KRAS^G12D^ proteins via exosomes, which are uptaken by AGER expressed on macrophages, inducing differentiation of macrophages from a M1-like phenotype to a M2-like tumor-supportive phenotype via STAT3-dependent fatty acid oxidation [123]. Moreover, ferroptotic cancer cells release excess fatty acids into the TME, effectively inhibiting CD8^+^ T and NK cell efficacy and cytokines secretion, such as IFN-γ, tumor necrosis factor α (TNF-α) and peroxisome activator receptor-γ (PPAR-γ). Fatty acids bind to CD36 on T cells triggering ferroptosis, which limits the anti-tumor activities of T cells by downregulation of the release of cytotoxic cytokines IFN-γ and TNF-α [124, 125]. Decreased production of IFN-γ and increased expression of the PPAR-γ was seen in NK cells of patients with DLBCL and in an Eµ-myc lymphoma model with increased iron metabolism [126]. These findings highlight a dynamic interplay between cancer cells and their TME via regulation of ferroptosis **(**Fig. 4**)**. Hence, targeting T cell-mediated ferroptosis represents a new approach in immunotherapy, and the combination of ferroptosis modulation and immunotherapy is a potential strategy for cancer treatment. However, the mechanisms of TME and ferroptotic lymphoma cell interaction during lymphoma progression remain to be elucidated.

## Targeting ferroptosis has therapeutic potential in various types of lymphoma

The currently available cancer treatment methods do not effectively resolve the issue of inherent drug resistance in cancer cells. Numerous studies have shown that ferroptosis plays a practical role in eliminating cancer cells and suppressing tumor growth in lung cancer [127], glioblastoma [128], colorectal cancer [129], pancreatic cancer [130], and prostate cancer [131]. Ferroptosis has also been found to play important roles in lymphoma. In particular, various lymphoma-related signaling pathways crosstalk with ferroptosis.

Biological markers associated with clinical outcomes identified through the analysis of samples (e.g., blood, saliva, urine, feces, and/or tumor tissue) guides precision medicine. BODIPY^TM^ 581/591 C11 (Table 2), a fluorescent tracer, monitors lipid peroxidation products in live cells and tissues to measure ferroptosis in vivo [132]. In addition, 3F3 anti−ferroptotic membrane antibody (3F3-FMA), a selective ferroptosis-staining reagent binding to TFR1, has been identified as a histopathological biomarker for ferroptosis [38]. Ferroptosis-related proteins GPX4, HADHB, and SECISBP2 can be prognostic biomarkers of lymphoma [49, 133, 134]. Some ferroptosis-related agents for clinical assessment were listed in Table 1. Moreover, the latest developments in detection technologies, such as small-molecule fluorescent probes [135], enhanced magnetic resonance imaging-guided therapy [136], CRISPR screens and metabolomics [27, 137], may be useful for ferroptosis-directed treatments. Such painstaking efforts will require collaborative and close multidisciplinary cooperation for translation into clinical practice.

**Table 2:** Ferroptosis-related detection technologies in cancer and lymphoma.

Ferroptosis−related application	Clinical relevance	Disease or models studied	Assay	Clinical information	Ref
BODIPY^TM^ 581/591 C11	Ferroptosis measurement	Human fibrosarcoma cell line	Flow cytometry	BODIPY 581/591 C11, a fluorescent indicator, tracks lipid peroxides in live cells.	[132]
3F3 anti−ferroptotic membrane antibody 3F3 − FMA	Histopathological biomarker	B cell lymphoma mouse xenograft	Flow cytometry, Immunofluorescence	3F3 − FMA, an anti−TFR1 antibody, inhibits iron uptake and specifically stains cells undergoing ferroptosis.	[38]
GPX4	Prognosis	DLBCL	Immunohistochemistry	GPX4 was expressed in 35.5% (33/93) DLBCL patients and associated with poor OS (*P* = 0.0032) and PFS (*P* = 0.0004).	[49]
HADHB	Prognosis	DLBCL	Immunohistochemistry	HADHB was overexpressed in 68% (87/128) DLBCL patients and was an independent predictor for poor prognosis (*P* = 0.001).	[134]
SECISBP2	Prognosis	DLBCL	Immunohistochemistry	SECISBP2 was expressed in 45.5% (75/165) DLBCL patients and associated with poor OS (*P* = 0.006).	[133]

Recently, drugs targeting ferroptosis have displayed pre-clinical efficacy through two main approaches: (1) inhibiting antioxidant mechanisms such as the SLC7A11–GSH–GPX4 axis, and (2) inducing ferroptosis by increasing iron uptake, oxidation, and the LIP level modulated by LTF, SLC40A1, TFRC, and NCOA4.

**Fig. 5:**
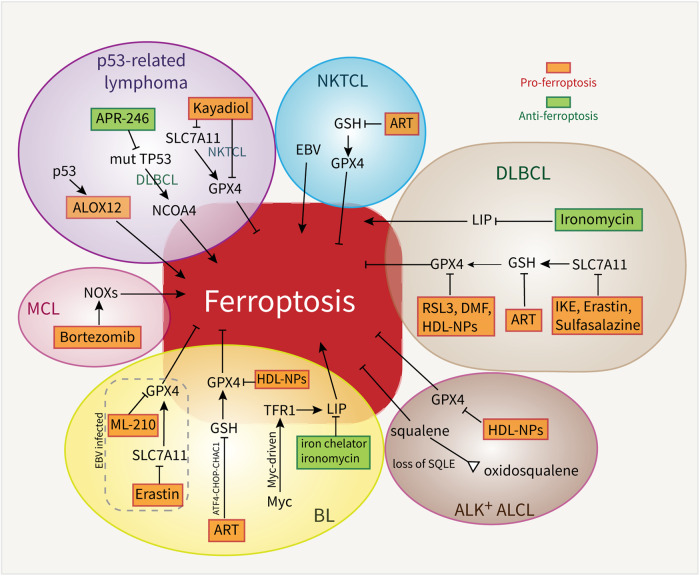
Therapeutic strategies for targeting ferroptosis in lymphoma. Inducers and inhibitors of ferroptosis are depicted in red and green, respectively. ART Artesunate, ALCL anaplastic large cell lymphoma, BL Burkitt lymphoma, DLBCL diffuse large B cell lymphoma, DMF dimethyl fumarate, HDL-NPs high–density lipoprotein-like nanoparticles, IKE imidazole ketone erastin, MCL mantle cell lymphoma, NKTCL extranodal natural killer/T cell lymphoma, SQLE squalene monooxygenase.

Current research on ferroptosis in lymphoma has focused on targeting ferroptosis-specific molecules to increase the sensitivity of lymphoma cells to ferroptosis, thereby suppressing the growth and progression of lymphoma. In this section, we further discuss the relationships between ferroptosis and various types of lymphoma and summarize the ferroptosis-modulating agents and molecular targets in different types of lymphoma (Table 1 and Fig. 5).

### DLBCL

DLBCL is the most common malignant lymphoma accounting for ~30% of NHL, and is characterized by biological heterogeneity [138]. Abnormal iron homeostasis and lipid peroxidation are involved in the carcinogenesis and progression of DLBCL. Serum ferritin and transferrin levels can reflect cellular iron levels, and several anti-TFR1 and malondialdehyde antibodies [38] have been identified as effective reagents to selectively stain ferroptotic cells in tissues and multiple cell culture contexts including DLBCL cells [38].

A Japanese study group found that in a cohort of 93 patients with DLBCL, 35.5% of cases expressed GPX4 and had significantly worse OS and PFS than GPX4-negative cases [49]. However, treatment was heterogeneous in this cohort (47 R-CHOP and 46 CHOP cases). Later, this study group investigated SECISBP2 that regulates GPX4 translation in 165 DLBCL patients treated with R-CHOP, and HADHB involved in fatty acid beta oxidation in 128 DLBCL patients (treatment unknown), and also found significant unfavorable prognostic effects in DLBCL [133, 134] (Table 2). Some other researchers constructed ferroptosis-related risk score models for prognostic prediction (Table 3) based on transcriptional expression of ferroptosis-related genes available in the Gene Expression Omnibus (GEO, including our deposition GSE31312) and The Cancer Genome Atlas (TCGA) databases [96, 139–142]. In these studies, high-risk scores were consistently associated with significantly shorter survival; and in one study risk scores also correlated with simulated dosing response of DLBCL to doxorubicin [141]. However, there is much disparity in these risk models (Table 3), likely caused by the differences in the bioinformatics methods and cohorts employed for model construction (for example, models were based on clinical results in the overall cohorts of GSE87371 with heterogeneous regimens; GSE11318 and GSE57611 with CHOP-like regimens; and GSE10846 with CHOP-like and R-CHOP-like regimens, respectively). Furthermore, a study estimated enrichment scores of immune cells and immune inhibitory receptors by single-sample gene set enrichment analysis, and found that high ferroptosis-related risk scores were correlated with immunosuppressive cell infiltration (regulatory T cells and macrophages) and expression of immune checkpoint molecules (CTLA-4, PD-L1, LAG-3, and TIM-3) thus immunosuppressive TME [139]. Another study estimated immune infiltrate using CIBERSORT and found that high ferroptosis-based risk score group had higher infiltration levels of activated CD4 memory T cells, resting NK cells, M2 macrophages, and neutrophils, whereas lower levels of follicular helper T cells, activated NK cells and M0 macrophages [141]. Moreover, ferroptosis-related risk score can predict resistance to ibrutinib in DLBCL cells [139].

**Table 3:** Prognostic models based on expression of ferroptosis-related genes in lymphoma.

Cancer type	Ferroptosis-related gene signature	Clinical relevance	Prognostic risk scoring model (expression of genes)	Ref
DLBCL	*ALAS1, HIF1A, LRP2*	Good prognosis		[96]
	*HMOX1, HMOX2, HFE, ISCA1, MSCP, PPOX, STEAP1, TMPRSS6*	Poor prognosis		
	*GSS, HMOX1, PEBP1*	Good prognosis	Risk Score= 0.053×CP + 0.193×FTH1 + 0.210×GPX4 − 0.219×GSS − 0.208×HMOX1 + 0.397×HSBP1 + 0.058×HSPB1 + 0.057×LAMP2 − 0.185×PEBP1 + 0.106×SAT1 + 0.045×SAT2.	[139]
	*CP, FTH1, GPX4, HSBP1, LAMP2, SAT1, SAT2*	Poor prognosis		
	*UBC, CAPG, EPAS1, ACVR1B, GABARAPL1, TFAP2C, AKR1C3*	Good prognosis	Risk Score= −0.006906×DRD4 − 0.1427×TFAP2C − 0.03086×AKR1C3 + 0.08295×CHAC1 + 0.2237×ULK2 + 0.1026×CXCL2 − 0.2478×GABARAPL1 + 0.07055×TRIB3 − 0.01005×CYBB + 0.4483×IREB2 − 0.02139×EPAS1 + 0.1521×MT1G + 0.2654×ATG3 − 0.06966×CAPG + 0.4052×ATF4 + 0.03599×UBC.	[141]
	*MT1G, IREB2, TRIB3, ATF4, ATG3, CHAC1, ULK2*	Poor prognosis		
	*LAMP2, CAPG, NOX4, ACSF2, HAMP, MUC1, TFAP2C, NOX1, FTL, HIF1A, FTH1, AKR1C3, GABARAPL2, CRYAB, GLS2*	Good prognosis	Risk Score= −0.154587331×CAPG − 0.256381293×HAMP − 0.185050076×NOX4 + 0.263432584×SLC1A5.	[142]
	*PCK2, HMGCR, MAPK8, PEBP1, SCD, NFS1, GSS, SQLE, TP53, ALOX12B, SLC1A5, ULK2, TRIB3*	Poor prognosis		
	*ZEB1, NFE2L2, LAMP2, HIF1A, FH, CXCL2*	Good prognosis	Risk Score= −0.05455544×HIF1A − 1.87952976×NFE2L2 − 0.09480619×FH − 0.34575386×LAMP2 − 0.43828031×ZEB1 + 0.16447965×NGB + 0.47117607×PSAT1 − 0.20840707×CXCL2.	[140]
	*PSAT1, NGB*	Poor prognosis		
	*STEAP3*	Good prognosis		[160]
CLL	*AKR1C3, CXCL2, EPAS1, FTH1, IREB2, JDP2, PTGS2, SETD1B, SIRT1, SLC1A5, SP1, TLR4, VEGFA*	Good prognosis	Risk Score= −0.1930×AKR1C3 + 0.1751×BECN1 + 0.0897×CAV1 + 0.5965×CDKN2A − 0.0544×CXCL2 + − 0.1560×JDP2 − 0.3307×SIRT1 + 1.0947×SLC1A5 − 0.1027×SP1.	[156]
	*AIFM2, BECN1, CAV1, CDKN2A, GABARAPL2, HRAS, PEBP1, SLC1A5, VDAC2*	Poor prognosis		
	*PRKAA1, HELLS, FANCD2, CDKN2A*	Good prognosis	Risk Score= 0.14×TP63 + 0.45×STEAP3 + 0.79×NQO1 + 0.14×ELAVL1 − 0.11×PRKAA1 + −0.91×HELLS − 0.16×FANCD2 − 0.41×CDKN2A.	[157]
	*TP63, STEAP3, NQO1, ELAVL1*	Poor prognosis		

DLBCL is susceptible to GPX4-regulated ferroptosis. Inhibition of the GPX4 and system Xc^−^ results in accumulation of intracellular ROS, which enhances the sensitivity of DLBCL cells to ferroptosis [46, 143]. However, the clinical usage of GPX4 inhibitors is still limited to laboratory research due to their poor druggability [144]. High–density lipoprotein-like nanoparticles (HDL NPs) are a cholesterol-poor ligand that binds to scavenger receptor type B1 (SCARB1), the receptor for cholesterol-rich HDLs [19]. Treating cholesterol-addicted BL and GCB-DLBCL cells with HDL NPs activated a compensatory response upregulating de novo cholesterol biosynthesis genes which nearly abolished the expression of GPX4, resulting in ferroptotic death of BL and DLBCL cells in vitro and in vivo, as well in primary samples from patients with different types of lymphoma including DLBCL [19]. Similarly, treating previously identified cholesterol auxotrophic ALK + ALCL and histiocytic lymphoma cell lines (which depend on LDLR-mediated cholesterol uptake) [27] with HDL NPs also decreased GPX4 and induced ferroptosis [19].

IKE, an inhibitor of system Xc^−^ that is metabolically stable and suitable for in vivo applications, decreases glutathione and lipid peroxidation and slows tumor growth in mouse DLBCL xenografts with minimal side effects [57]. Schmitt et al. reported that dimethyl fumarate (DMF) induces lipid peroxidation and ferroptosis by inhibiting GSH and GPX4 expression, particularly in GCB-DLBCL [21]. These findings highlight the potential of lipid peroxidation as a therapeutic target for DLBCL.

In addition, targeting cellular iron metabolism has been proven to be an efficient approach. Ironomycin, which regulates iron homeostasis to decrease LIP levels, significantly reduces viability of primary DLBCL cells while presents lower toxicity in normal tissues and hematopoietic progenitors than conventional treatments [96]. Moreover, ironomycin exhibits synergistic effects with doxorubicin, BTK inhibitors, and Syk inhibitors [96]. APR-246, a p53 mutant reactivator, can induce ferroptosis in DLBCL cells by upregulating NCOA4 expression [91]. This in turn results in breakdown of ferritin and cellular LIP level decrease in a TP53-dependent manner. Artesunate, a semisynthetic derivative of artemisinin, can downregulate the expression of GPX4 and induce apoptosis, cell cycle arrest, autophagy and ferroptosis by impairing the STAT3 signaling pathway in DLBCL cells [145].

These studies indicate that ferroptosis is a major mechanism involved in the pathophysiology of DLBCL [46, 49, 57], concomitant with excessive ROS accumulation and lipid peroxidation. These findings added a new molecular mechanism underlying the pathogenesis of DLBCL and may aid the development of new therapies to induce RCD in DLBCL (Table 1 and Fig. 5) and new combination treatment strategies. However, validation studies and the heterogeneity in response to treatment need further investigation.

### Mantle cell lymphoma

MCL comprises 5% to 10% of NHL, characterized by genetic alterations involving p53, cyclin-dependent kinase (CDK) 4, CDKN2A, MYC, BCL-2, BCR, and nuclear factor (NF)-κB [146]. MCL cells require large quantities of iron to meet their high proliferation requirements, rendering them more vulnerable to iron deprivation [147]. Thus, iron chelators have been shown to exert cytotoxic effects in MCL cells [148]. Moreover, bortezomib induces mitochondrial depolarization and ROS generation by upregulating the BH3-only protein Noxa in MCL cell lines and primary MCL cells [149], suggesting that ferroptosis-targeted combination strategy could be further explored.

### Burkitt lymphoma

BL is a highly aggressive B-cell NHL that is associated with EBV and was the first tumor to be reported to harbor a chromosomal translocation of *MYC* [150, 151]. A recent study found that different lipid peroxidation byproducts are generated at different stages of EBV transformation. Detoxification of lipid peroxidation productions and ROS by GPX4 and glutathione, respectively, is required for the sustained growth of Burkitt-like B cells. Interference in redox defense mechanisms induces ferroptosis in the earlier stages of EBV transformation or in BL cells (latency I) [152]. This phenomenon highlights the potential of ferroptosis inducers as a therapeutic approach for EBV-associated BL. Wang et al. reported that ART, as a ferroptosis inducer, enhances ferroptosis and suppresses proliferation by activating the ATF4–CHOP–CHAC1 pathway in BL in vitro and in vivo [153]. MYC regulates ferroptosis by facilitating the uptake of iron into the LIP. Thus, iron chelators and ironomycin may be effective treatments for *MYC*-driven lymphoma [96]. Moreover, deletion of *TP53* dramatically accelerates the growth of Myc-induced lymphoma cells; however, blockade of ALOX12 inhibits p53-mediated ferroptosis [86]. Therefore, ALOX12 is a promising molecular target that can inhibit the development and progression of Eμ-Myc lymphoma by regulating the expression and function of p53.

### Small lymphocytic lymphoma/chronic lymphocytic lymphoma

SLL/CLL is characterized by the peripheral accumulation of abnormal CD5 + B cells [154] and remains incurable [155]. Ferroptosis-related prognostic score models have been developed to predict the survival of patients with CLL (Table 3) [156, 157]. These models can be used to assess the response of patients with CLL to fludarabine, cyclophosphamide, and ibrutinib, as patients with low risk scores are more likely to benefit from these treatments and exhibit better OS [157]. However, a comprehensive molecular understanding of ferroptosis in CLL is lacking as few studies have reported ferroptosis in CLL.

### Extranodal natural killer/T cell lymphoma

NKTCL is a highly aggressive type of NHL with an aggressive clinical course and is closely associated with EBV infection [158], which poses treatment challenges. A study showed that kayadiol, a natural compound extracted from *Torreya nucifera*, induces accumulation of ROS and ferroptosis by targeting the SLC7A11–GSH–GPX4 axis [90]. Kayadiol effectively killed NKTCL cells but not healthy lymphocytes and hence presents a potential treatment option for NKTCL, particularly in patients who develop chemoresistance.

## Conclusions and perspectives

Similar to other types of RCD, ferroptosis plays an essential role in the occurrence and progression of lymphoma. Multiple physiological processes regulate ferroptosis, such as iron metabolism, lipid metabolism, mitochondrial metabolism, and the TME. Although various studies have confirmed the role of ferroptosis in cancer development, the detailed role in lymphoma remains unclear. Ferroptosis is a highly complex process whose role in tumor initiation and progression is context-dependent. Owing to the heterogeneity of lymphoma, ferroptosis signaling pathways and molecular mechanisms are poorly understood. More in-depth insights into the mechanisms of ferroptosis are needed to develop efficient ferroptosis-inducing strategies that can be used for lymphoma treatment.

Furthermore, there is crosstalk between ferroptosis and other RCD pathways as it involves similar gene mutations and protein alterations, making it difficult and all the more necessary to distinguish ferroptosis from non-ferroptotic cell death. To date, no specific biomarker has been identified for ferroptosis. The development of sensitive and reliable specific biomarkers or assays may promote research on ferroptosis in lymphoma and guide the clinical application of ferroptosis-based therapies for lymphoma. In addition, components of the TME are susceptible to ferroptosis, which in turn can influence anti-tumor immune responses. Therefore, balancing the sensitivity of cancer, cytotoxic T cells, and immunosuppressive cells to ferroptosis remains a major hindrance. Exploring the mechanisms underlying these relationships is indispensable to address this issue.

Finally, although many drugs have been reported to induce ferroptosis in cancer and lymphoma cells in preclinical studies, none have entered clinical trials. Ferroptosis-targeted approaches face challenges in clinical application such as the systemic toxicities [159], and future studies need identify novel targets for therapeutic development, such as SCARB1 [19] and LDLR [27]. The effectiveness of HDL NPs observed in both B and T cell lymphomas may suggest common metabolic vulnerability exists in different lymphoma subtypes making targeted therapy appealing. We hope our review on ferroptosis studies provides a comprehensive overview and offer a new research direction for lymphoma therapeutic development for future investigations.

## Data Availability

Not applicable.
