# Correction: Mechanisms of ferroptosis and targeted therapeutic approaches in lymphoma

**DOI:** 10.1038/s41419-023-06393-9

**Published:** 2024-01-09

**Authors:** Tiantian Yu, Zijun Y. Xu-Monette, Li Yu, Yong Li, Ken H. Young

**Affiliations:** 1 Hematopathology Division and Department of Pathology, Duke University Medical Center, Durham, NC USA; 2 Department of Hematology and Oncology, The Second Affiliated Hospital of NanChang University, Nanchang, China; 3 Department of Medicine, Baylor College of Medicine, Houston, TX USA; 4 Duke Cancer Institute, Durham, NC USA

**Keywords:** B-cell lymphoma, Cell death

Correction to: *Cell Death* and *Disease* 10.1038/s41419-023-06295-w, published online 25 November 2023

In page 5 of published pdf, in the 8th line of the second paragraph on the left side, there is an extra word “for” which need to be deleted. After correction, that sentence is: “Different from the induction of ferroptosis by erastin or GPX4 inhibitors requiring ACSL4, p53-mediated ferroptosis under ROS stress requires ALOX12 and p53 activation [86].”

Also, in page 5 in the 12th last line on the right side, there is a spelling mistake, “cyst(e)in” should be “cyst(e)ine”. After correction, that sentence is: “The MYC family member MYCN was reported to mediate cysteine addiction, and make the fast proliferating cells vulnerable to cyst(e)ine depletion, inducing MYCN-dependent ferroptosis [95].”

The original article has been corrected.

